# Cognitive Functioning, Health Screening Behaviors and Desire to Improve One’s Health in Diabetic versus Healthy Older Women

**DOI:** 10.9734/JAMMR/2017/34173

**Published:** 2017-08-16

**Authors:** Luciana Laganá, Kimberly Arellano, David Alpizar

**Affiliations:** 1 Department of Psychology, California State University Northridge, 18111 Nordhoff Street, Northridge, CA 91330-8255, USA; 2 Department of Health Sciences, California State University Northridge, 18111 Nordhoff Street, Northridge, CA 91330-8285, USA; 3 Department of Educational Leadership, Sports Studies, Educational/Counseling Psychology, Washington State University, Pullman, 14204 NE Salmon Creek Ave, 98686, Pullman, WA 99163, USA

**Keywords:** Diabetes, older women, cognitive functioning, cancer, health screening

## Abstract

**Aims:**

To attempt to fill a gap in the literature on diabetic versus healthy older women on desire to improve one’s health, health screening behaviors, and cognitive health.

**Study Design:**

Between-subjects design.

**Place and Duration of Study:**

Department of Psychology, California State University Northridge, between July 2013 and June 2015.

**Methodology:**

In this preliminary study, we compared 30 diabetic older women to 42 healthy older women (i.e., respondents who reported having no physical illnesses and not taking any medications) on: desire to improve their health (hypothesized as being higher in the diabetes group), receiving mammograms and regular health screenings (analyzed without any hypotheses, due to the lack of evidence on this topic), as well as cognitive functioning (hypothesized as lower in the diabetes group, based on prior research findings). Participants (*N*=72, *mean age*=69.29, *SD*=6.579, a*ge range*=50–90) were multiethnic, non-institutionalized women over the age of 50 residing in Los Angeles County who completed our research packet. The latter contained the first author’s demographics list and her original structured interview protocol on older women’s health, as well as the well-known Mini-Cog.

**Results:**

The results of an Analysis of Variance (ANOVA) showed that, as hypothesized, diabetic women desired to improve their health more than the women in the control group [*F* (1,70)=11.87, *p*<.05, *η^2^*=.15]. Additionally, upon implementing Chi-square analyses, we discovered that diabetic respondents were significantly more likely to receive mammograms [*X^2^*(1)=5.87, *p*<.05] and general health screenings [*X^2^*(1)=4.51, *p*<.05] than healthy women. Moreover, in contrast with prior literature’s findings, cognitive health in the diabetic group obtained marginal significance in an ANOVA as being better than the cognitive health of the control group [*F*(1,68)=3.30, *p*=.06, *η^2^*=.05].

**Conclusion:**

We have established a significant relationship between diabetes and a) desire to improve one’s health and b) health screening behaviors, as well as c) cognitive impairment (at a marginally significant level) among diabetic versus healthy women. This has important clinical and public health implications. Although the findings of prior research suggest that diabetic older women often experience impaired cognitive performance compared to healthy older women, our marginally significant results showed that the opposite is true, at least in our ethnically diverse sample of modest size. Moreover, we found that diabetic older women desired to improve their health significantly more than healthy women and pursued cancer screenings and general health screenings more than their healthy counterpart. The limited size of our sample does not allow for generalizations of our findings. Additional research with larger samples is definitely needed to investigate these topics further.

## 1. INTRODUCTION AND THEORETICAL FRAMEWORK

The purpose of this study was to attempt to fill a gap in the literature by comparing diabetic older women to healthy older women regarding their desire to improve health, health screening compliance, and cognitive function. Older adults represent one of the fastest growing age groups in the United States (U.S.), with individuals age 65 or older numbering 43.1 million in 2012 [1]. Between 2012 and 2050, the U.S. will experience considerable growth in its older population [2]. The number of people over the age of 65 is expected to double to more than 70 million by the year 2030, when it will equate to approximately 21% of the U.S. population [1]. This means that nearly one in five U.S. residents will be 65 or older in 2030 [3]. Scholars [4] have also estimated that the percentage of individuals age 65 and older is expected to grow from 15% to 24% between 2014 and 2060. Additionally, women represent the fastest growing segment of the older population, making the aging population in the U.S. composed primarily of women [5]. Thus, it is important to identify factors that could improve or at least preserve older women’s quality of life.

The health status of the older U.S. population has improved over the years both in terms of living longer and remaining functional. In 2011, persons reaching age 65 had an average life expectancy of an additional 19.2 years (20.4 years for women and 17.8 years for men [6]); however, a longer life does not necessarily mean a healthier one. Age is the most consistent risk factor of illness and death across the total population [7]. Older adults are at high risk for developing chronic illnesses and related disabilities. These chronic conditions include diabetes mellitus, arthritis, congestive heart failure, and dementia. It has been estimated that more than 37 million older adults will have to manage more than one chronic condition by 2030 [8]. Consequently, many older adults will likely experience hospitalizations and nursing home admissions. They may also lose the ability to live independently at home. Moreover, chronic conditions remain the leading cause of death among older adults [9].

In the present study, we investigated the health behaviors, desire to improve one’s health as well as the cognitive functioning of diabetic older women as opposed to healthy older women who do not take any medications and report having no illnesses. We chose as a guiding theoretical framework for our study the classic Health Belief Model by Hochbaum [10], which is useful in explaining self-care activities such as diabetes management recommendations, and has a focus on behavior related to the prevention of the disease. Very briefly, the foundation of this model is that individuals will take action to prevent, control, or treat a health problem if they perceive the problem to be severe in nature, based on their beliefs and attitudes towards health and treatment. However, with advanced age, attitudes and beliefs about health and illness can become negative, yet the perceived severity of the disease often decreases [11]. To target the care and effective management of diabetes in older adults, it is crucial to recognize patients' beliefs and attitudes about behaviors towards their health and illness [12], in line with the Health Belief Model and as done in the present investigation. Thus, research is needed to clarify attitudes and beliefs towards health and health practices such as receiving health screening in healthy older populations as well as in patient populations such as diabetic older women. In the following paragraphs, we have provided a brief summary of the literature on our study’s variables of interest.

### 1.1 Diabetes

Diabetes mellitus (DM), commonly referred to as diabetes, is a group of chronic diseases associated with high levels of blood glucose resulting from deficits in insulin production, insulin action, or both. Diabetes affects an estimated 23.6 million people in the U.S. (7.8%) and only 17.9 million are aware of it [13]. Type 1 and type 2 diabetes are the most common forms of diabetes [14]. Type 1 diabetes, formerly called juvenile or insulin dependent diabetes, is experienced by 5–10% of diabetic patients and typically develops in childhood or early adulthood [15]. In most patients, the age of onset is younger than 30 years. Type 2 diabetes is characterized by insulin resistance and typically occurs later in life; its incidence increases with age, with the risk rising at 45 years old or older [16]. The current prevalence of diabetes in the U.S. peaks at 10–20% around 70 years of age [15]. Additionally, a sedentary lifestyle can increase the risk of type 2 diabetes [17]. Approximately 80% of people with type 2 diabetes are overweight or obese [16]. Moreover, diabetes is associated with an increased risk for a number of serious, sometimes life-threatening complications; currently, it affects 23.6 million people in the U.S. and is the 7^th^ leading cause of death [13]. Diabetes increases the risk of heart disease by 2 to 4 times and lowers life expectancy by up to 15 years [18]. Furthermore, it is the leading cause of kidney failure, lower limb amputations, and adult-onset blindness [13,19]. As the rate of diabetes continues to increase, as many as 1 in 3 American adults will have diabetes in 2050 if present trends continue [20].

### 1.2 Diabetes in Older Adults

Diabetes is highly prevalent among older adults. According to the American Diabetes Association, approximately 8.6 million adults age 60 or older were affected with diabetes in 2002 [14]. By 2050, the largest increase (336%) in prevalence of diagnosed diabetes will be in individuals 75 years or older [21]. Older adults with diabetes are at especially high risk for developing cardiovascular complications, kidney damage, vision problems, neuropathy, foot problems, and cognitive impairment [22] and make up the largest population seeking care for diabetes [23]. Although the prevalence of diabetes is comparable for both sexes in most populations, among women, diabetes generally has a more devastating impact and is more difficult to control and affects up to 18% of women 65 years or older [24].

### 1.3 Older Adults and Health Screenings

Older adults are less likely to use preventive health services than younger or middle-aged adults [25]. Their rates of cancer screenings, flu shots, mammograms, and pap smears are typically below recommended levels [26]. Researchers have called for routine preventive health services for older adults including immunizations, screening tests, and counseling to prevent the onset or progression of disease and disability in order to best maintain the health of older adults [27]. Older adults who pursue clinical preventive services and practice healthy behaviors are more likely to remain healthy and functionally independent [28]. Clinical preventive services can help prevent chronic disease, reduce associated complications, and lower functional limitations [29]. However, less than half of adults age 65 or older report being up-to-date on these services [30]. In a 10-year agenda for improving the Nation’s health, Healthy People 2020 recently added as a national objective to increase by 10% the proportion of men (from 46.3% to 50.9%) and women (from 47.9% to 52.7%) age 65 or older who are up to date on the core set of clinical preventive services [30]. These core preventive services include influenza and pneumococcal vaccinations, lipid disorders screening, colorectal cancer screening, diabetes screening and, for women, breast cancer screening.

In general, individuals with known risk factors for diabetes or those with symptoms of possible diabetes complications are targets for screening programs. Given that diabetes is very common in older populations and is associated with significant mortality and morbidity if left untreated, it is recommended that all individuals over the age of 45 should be considered as candidates for screening [31]. Early diagnosis and treatment can delay health problems and prevent complications. Older adults age 65 or above and in good health should be screened for diabetes every three years [32]. Despite regular checkups, fewer than half of adults age 65 or older are up to date with clinical preventive services, including diabetes screening [33]. Unfortunately, approximately a third to half of diabetes patients are undiagnosed, and about a third of type 2 diabetes patients are diagnosed with complications [34]. Furthermore, in data illustrating rates of older adults not receiving preventive health services, 31% of adults age 65 or older without diagnosed diabetes reported not receiving a test for high blood sugar or diabetes within the past three years [13]. To our knowledge, there are no studies on the health screening practices of diabetic versus healthy older women, thus the present study will shed some light on this medical topic.

### 1.4 Diabetes and Breast Cancer

In the U.S., breast cancer is the most commonly diagnosed form of cancer for women and is one of the leading causes of women’s morbidity and mortality, regardless of ethnic background [35]. Moreover, advancing age is the single most critical risk factor in the development of breast cancer, as one in 10 women over the age of 65 will develop breast cancer [36]. Diabetes has been associated with an increased risk of several types of cancers, including breast cancer. At present, up to 16% of patients with breast cancer who are older than 65 also have diabetes mellitus [32], thus the incidence of both breast cancer and diabetes is quite high in older women. Because diabetes increases the risk of breast cancer and breast cancer mortality, adequate breast cancer screening in older women with diabetes is important. Mammogram screening allows for the early detection of malignancies and has been shown to reduce breast cancer mortality by up to 40% in women between 50 and 69 years of age [37]. Based on these findings, the U.S. Preventive Services Task Force recommends mammogram screening, with or without clinical breast examination, every 1 to 2 years for women age 40 or older [38]. However, the current literature suggests that a significant number of women eligible for screening are not getting regular mammograms. Additionally, mammogram screening rates are lower for women with diabetes, despite more visits to the physician [39]. Similarly, among older diabetic women, the incidence of non-diabetes-related preventive services including mammography screening is lower compared to non-diabetic women [40]. These findings suggest that, in spite of the complexity involved in diabetes care, routine preventive care such as cancer screening is often neglected.

### 1.5 Diabetes and Cognitive Function

In addition to diabetes-related complications affecting the kidney, eyes, and peripheral nervous system, the brain of diabetic patients is also affected by the illness. Both older age and diabetes are independently associated with an increased risk of cognitive dysfunction, but the risk is even greater for older adults with diabetes [41]. Considering the importance of self-management behaviors in the treatment of diabetes and the complexity involved in treatment regimens such as blood glucose testing, meal planning, and medication compliance, diabetic patients with cognitive dysfunction may experience difficulty managing their diabetes. Diabetes is associated with lower levels of cognitive function and greater cognitive decline among older women [24]. Research on diabetes as it relates to several aspects of cognitive function among community dwelling women (aged 70 to 78 years) has shown that women with type 2 diabetes a) perform worse than their counterparts on tests measuring cognitive function and b) are twice as likely to achieve a low cognitive score as those without diabetes [42]. Although diabetes may be indicative of cognitive dysfunction in women, there is a lack of studies on diabetic versus healthy older women regarding their cognitive health. Thus, discovering a significant relationship between diabetes and cognitive functioning among older women, as attempted in the present study, would have important clinical and public health implications.

### 1.6 Desire to Improve Health among Older Adults and the Health Belief Model

Older diabetic patients are expected to switch to a new lifestyle at the moment of diagnosis, have adequate diabetes knowledge and skills, and maintain a positive attitude to prevent complications and decreased quality of life as well as to successfully manage this serious illness. To target the care and effective management of diabetes in older adults, it is crucial to recognize patients' beliefs, behaviors and attitudes related to their health and illness [11], as done in the present study, in which we inquired about desire to improve one’s health. Indeed, the aforementioned Health Belief Model [10] is often used in the management of chronic diseases such as diabetes. The health beliefs of diabetic individuals are important factors in health related behaviors. The effective management and control of diabetes requires behavioral compliance including receiving regular health screenings.

Researchers who have used the Health Belief Model and have sampled diabetic patients have reported a significant correlation between people’s health related beliefs and attitudes about the disease as well as the behavioral compliance necessary for the treatment of diabetes [43–46]. However, there is a lack of literature focusing on and discussing the health beliefs of the older population in the U.S., especially of older diabetics. Furthermore, because with advanced age attitudes and beliefs about health and illness could understandably become more negative, this could in turn negatively impact older adults’ preventative health behaviors, including complying with medical regimens and pursuing health screenings.

To ensure diabetic patients’ adoption of positive health behaviors, the health beliefs and attitudes of these patients must be assessed. Thus, the study of attitudes and beliefs about diabetes is important. Yet, to our knowledge, there is no prior published literature examining the health beliefs or the desire to improve health among older diabetic women versus health older women. The growing older population of women from diverse ethnic backgrounds and the high prevalence of diabetes in older age make this research crucial, thus the present study fills a gap in the ethnogeriatric literature.

### 1.7 Hypotheses and Research Question

Desire to improve one’s health was hypothesized as being higher in the diabetes group. Conversely, cognitive function was hypothesized to be lower in the diabetes group, based on available research findings. Moreover, given the paucity of investigations in the area of diabetic older women’s health screening practices, we posed two research questions, namely whether receipt of regular health screenings and of mammograms in particular would have been higher among healthy or among diabetic older women.

## 2. MATERIALS AND METHODS

### 2.1 Sample

A total of 72 women (50 to 90 years of age) participated in this study, which was approved by the Institutional Review Board of California State University, Northridge (CSUN), as part of a larger federally funded study on older women’s quality of life. The research participants were non-institutionalized, community dwelling women living in Los Angeles County, California, an excellent location for gathering an ethnically diverse sample. Thirty participants self-reported having diabetes, and 42 self-reported not having any illnesses and not taking any medications. The characteristics of the sample are illustrated in Table 1. To summarize, age of the participants ranged from 50 to 90 (*SD=*8.175). The mean age was 69.29. The majority of the participants reported being married (52.8%). Over 50% of the population did not attend college. Moreover, about 25% of the sample had a high school diploma at best. The ethnic background of the sample was very diverse and mainly non-European-American. Concerning participants’ economic status, about half of the sample reported having an annual household income lower than $40,000.

### 2.2 Procedures

The present study took place between 2013 and 2015 and was conducted in full compliance with the ethical standards concerning research on human participants. Recruitment involved purposive sampling at places such as stores and parks as well as snowball sampling via participants’ referrals. Our participants were willing and able to engage in the study and had the right to withdraw from it at any time, as specified in the consent form. We only recruited women who met all the following criteria: a) being at least 50 years of age, in accordance with the definition of the start of older adulthood by agencies such as the Centers for Disease Control and Prevention [47]; b) being able to speak and understand English fluently (to minimize confounding our findings with levels of acculturation); and c) living in a non-institutionalized setting (to gather a community-dwelling sample). Research assistants (RAs) interviewed eligible participants for about 45 minutes to an hour. This was done in the comfort of participants’ homes or at community locations such as schools and libraries. Each woman was assigned a random number between 1 and 500 and the RA assigned to the data collection for the participant in question wrote this number on her research packet.

### 2.3 Variables Assessed and Corresponding Instruments

#### 2.3.1 Demographics

Participants’ demographic characteristics were quantified through utilization of a simple demographics list that was created by the first author. This list contains 10 items that inquire about respondents’ characteristics such as age, educational level, place of birth, employment status, marital status, and income.

#### 2.3.2 Interview protocol

This protocol was developed by the first author and included the following items that were of interest in the present study:

- Have you ever received a mammogram? If no, why not? If yes, when did you receive the last mammogram?
- Do you regularly receive health screenings? If no, why not? If yes, what types? (To investigate both general check-up and specific health screenings, including mammograms in particular).
- If you could, how much would you like to change your health, in order to feel that you are healthy? (This item was answered on a 0 to 5 Likert scale, with 0 = not important at all to 5 = important).

#### 2.3.3 The Mini-Cog

This measure is a simple screening tool that is widely used to detect cognitive impairment [48]. It only takes three minutes to administer it; this short administration time is an advantage when wanting to quickly screen for dementia in the older adult population. The Mini-Cog uses a three-item recall test for memory and a simple scored clock-drawing test (CDT). The CDT component of the Mini-Cog is often used to screen for cognitive impairment and allows clinicians to rapidly assess numerous cognitive domains including memory, language comprehension, visual-motor skills, and executive function and to obtain a visible record of both normal and impaired performance that can be tracked over time. Available empirical evidence supports this tool’s validity with ethnically diverse older adult populations as being just as strong as or even better than longer established cognitive screening tests for use with multi-ethnic populations [49]. Via using this measure, participants are assessed on three items and asked to repeat back and remember those items. Participants are then asked to draw a clock face with all of the numbers, and then draw in the hands of the clock to indicate a certain time. The clock drawing test in particular has been widely used in clinical neuropsychological practice [50]. After a participant has drawn the clock face, this person is asked to repeat back the three items that were previously mentioned. The test is scored as follows: Recall of 0 items indicates cognitive impairment. Recall of 1–2 items with an abnormal clock face indicates cognitive impairment. Recall of 1–2 items with a normal clock indicates no cognitive impairment. Recall of 3 items indicates no cognitive impairment. The clock face is considered normal if all numbers are present in the correct sequence and position, and the hands readably display the requested time.

### 2.4 Analytic Strategy

Our data met required assumptions (e.g., normality, absence of outliers and multi-collinearity) for the analyses. In our variables, only cognitive function had a few missing values, less than 2 percent. To handle this situation, we chose among a number of acceptable procedures (including mean imputation or deleting the cases); as suggested by Tabachnick and Fidell [51], we deleted these few cases. Next, we performed multiple statistical analyses to test some of our hypotheses using SPSS. We conducted two one-way Analyses of Variance (ANOVAs) to examine group differences on desire to improve health and cognitive function. For the cognitive variable, age could have been a confounding factor, given that cognitive decline is common as people age [52], thus we needed to test for significant age differences between the two groups. In this regard, a one-way ANOVA revealed that respondents with diabetes were not significantly different on age than the control participants [*F*(1,70)=1.538, *p*=.219]. Hence, age was not included as a control variable. To test our two research questions concerning health screenings, we conducted a set of two Chi-squares to examine group differences on receiving regular health screenings and receiving mammograms (both assessed via asking yes or no responses, to minimize fatiguing older women with extensive questioning). To evaluate the significance of each analysis, we chose *α* = .05. Also, we reported Cohen’s *d* for each of the analyses. Cohen’s *d* values were evaluated via following the traditional Cohen’s criteria [53]: small (2≤ *d* ≤.5), medium (.5≤ *d* ≤.8), and large (*d* ≥.8).

## 3. RESULTS

**Fig. 1:**
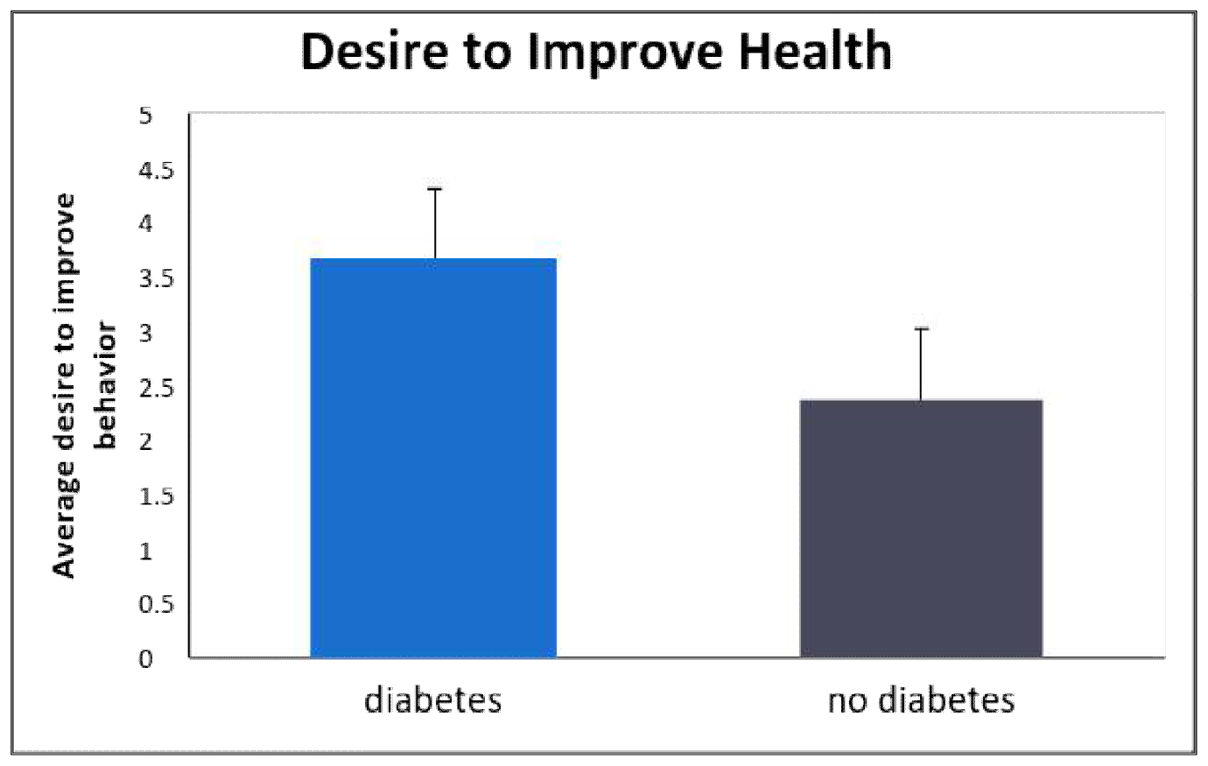
Graphic representation of desire to improve health results by group

**Fig. 2:**
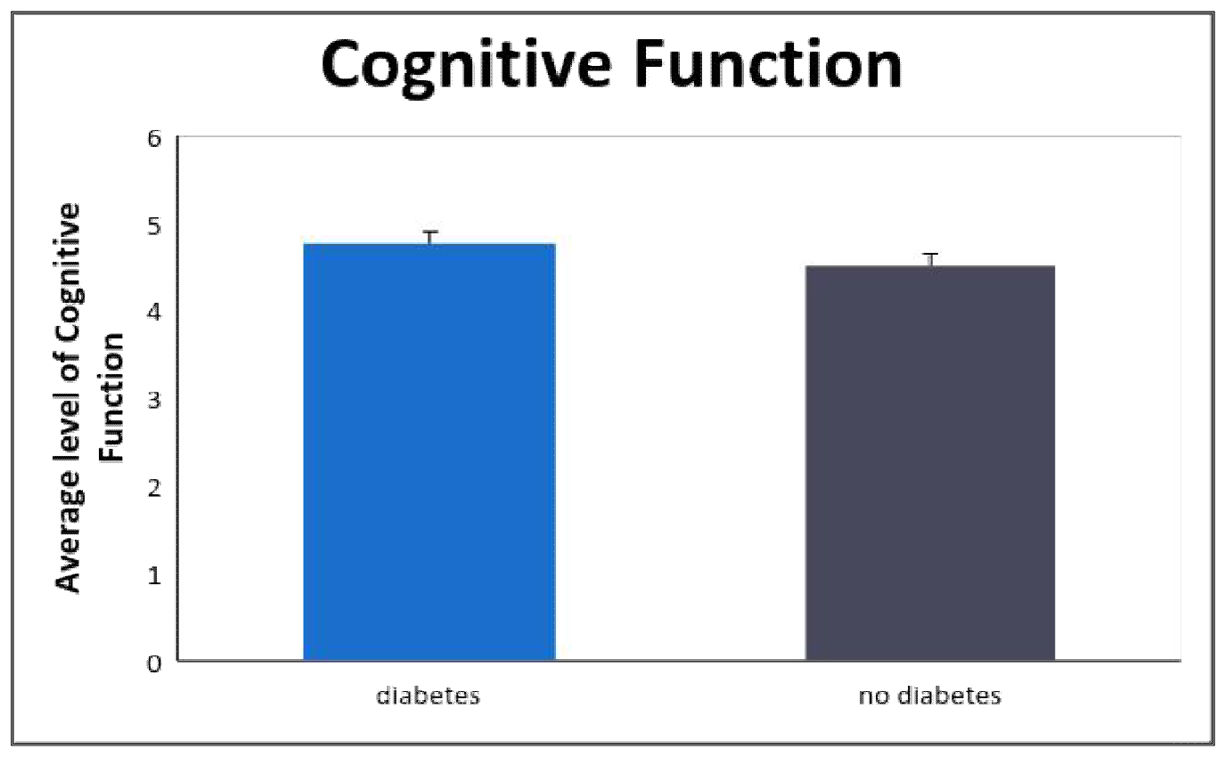
Graphic representation of cognitive functioning results by group

Table 2 displays the results of the two ANOVAs. This table shows that, for the first ANOVA, respondents in the diabetes group desired to improve their health significantly more than the control group [*F* (1,70)=11.87, *p*<.05, *η^2^*= .15, Cohen’s *d* = .85], as depicted in Fig. 1. The effect size was large. Concerning group differences in cognitive function, as illustrated in Fig. 2, we obtained marginal significance in the second ANOVA, with diabetic older women having better (*not worse*) cognitive functioning than their healthy counterpart [*F*(1,68)=3.30, *p*=.07, *η^2^*=.05, Cohen’s *d* =.44]. The effect size was small.

**Fig. 3:**
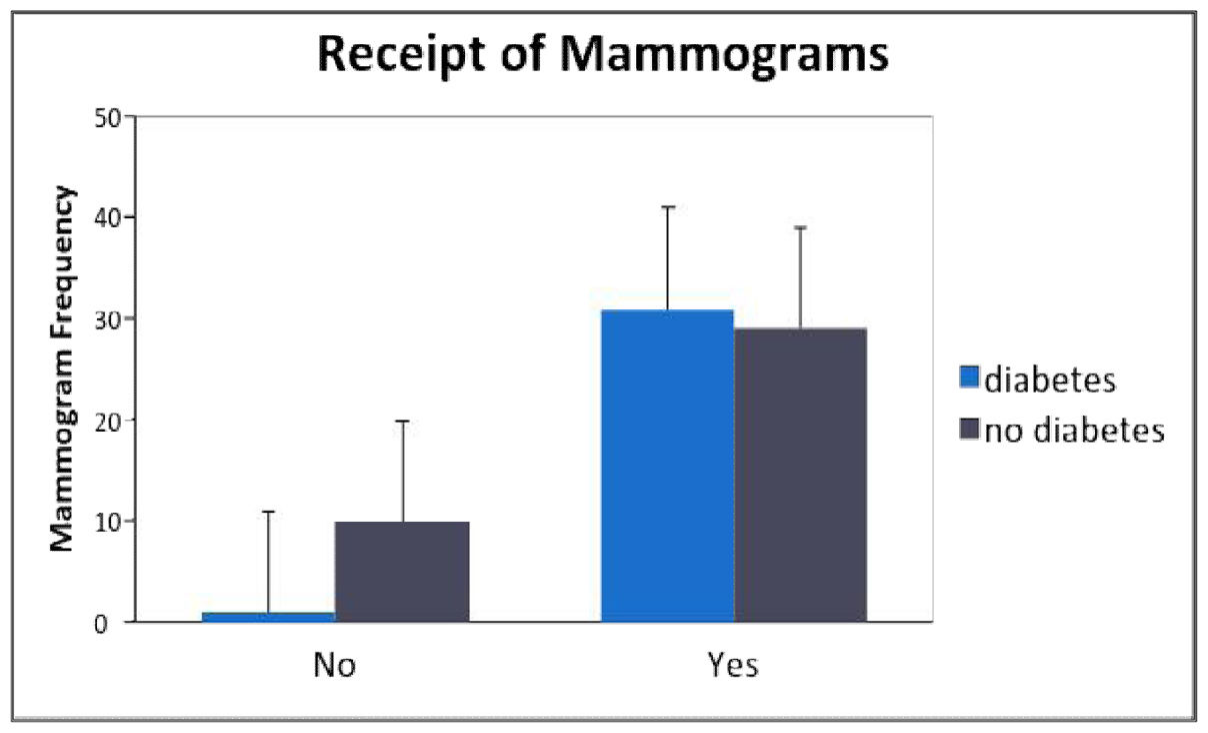
Graphic representation of mammogram results by group

**Fig. 4:**
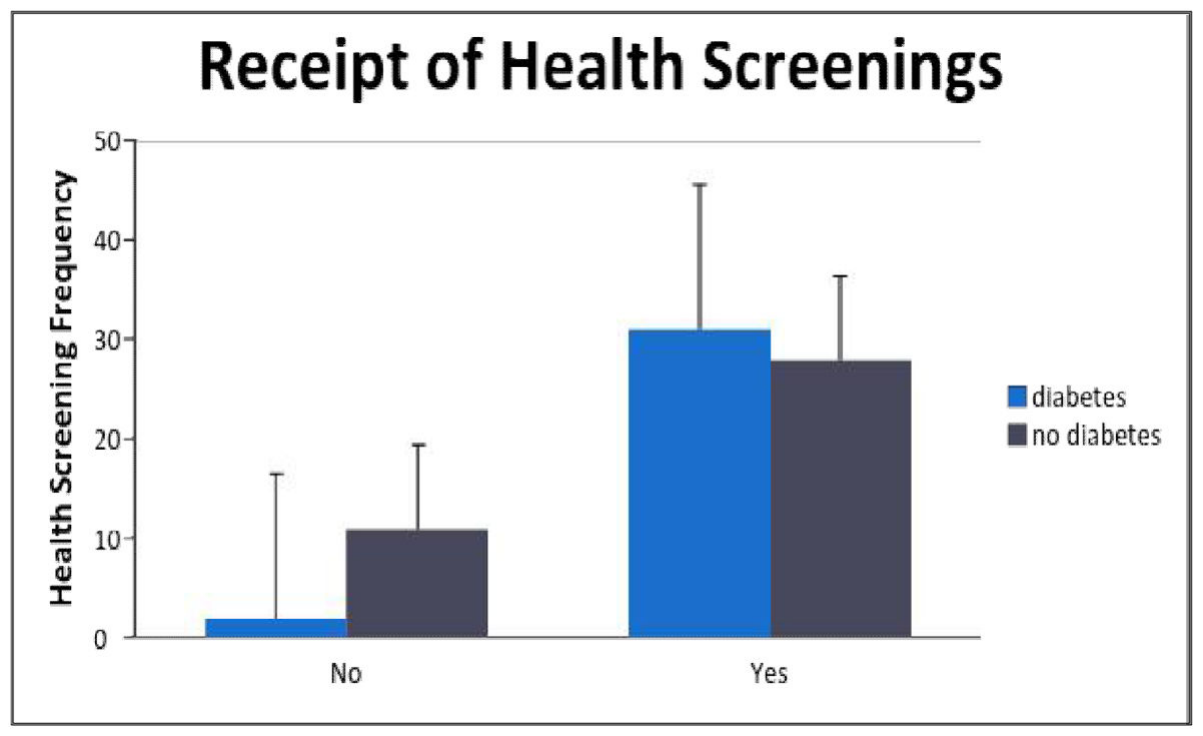
Graphic representation of general health screening results by group

Chi-square analyses were conducted regarding health status differences on health screenings. Results of the two Chi-square analyses are displayed in Table 3 and illustrated in Figs. 3 and 4. Interestingly, the diabetes respondents were significantly *more* likely to receive regular mammograms [*X^2^*(1)=5.87, *p*<.05, Cohen’s *d* = .60] as well as general health screenings [*X^2^*(1)=4.51, *p*<.05, Cohen’s *d*= .52]. Both effect sizes were medium.

## 4. DISCUSSION

In this study, we found that diabetic older women were significantly more likely to desire to improve their health, as expected, given their serious health condition. Also, they had better cognitive function at a marginal significance level. Perhaps, the diabetes group was receiving more health care that was reflected in better cognitive care but, to our knowledge, there is no prior empirical evidence supporting this finding in the literature. According to the results of previous studies, diabetes is associated with lower levels of cognitive function and greater cognitive decline among older women, but this was not the case in the present study. Considering the importance and complexity of self-management behaviors in the treatment of diabetes, lower cognitive function represents a significant threat to the ability of older women to follow health care advice and keep their diabetes in check. Our results, which are in need of duplication before drawing definite conclusions on this topic, suggest that adequate management of diabetes may help or prevent cognitive decline, at least to some degree.

Also, possibly due to consulting doctors on a regular basis for their health condition, diabetic older women were significantly more likely to receive mammograms as well as general health screenings. These results contradict those of previous studies stating that older diabetic women are less likely to pursue non-diabetes related preventive services such as general health and mammogram screenings. Indeed, in spite of the fact that researchers have suggested that routine non-diabetes-related preventive services are usually neglected by older diabetic women, as a new diagnosis of diabetes is made, older women undergo many tests and general health exams, which may include breast cancer screening with mammograms. Our interview questions did not include the date of diabetes diagnosis, thus we could not ascertain whether our diabetic respondents a) had received health screenings due to being recently diagnosed with diabetes and thus were assessed in their health more thoroughly than their counterparts, or b) regardless of the time of diagnosis, they were still making sure that their health was under control by pursuing regular health screenings, more so than the older women who were not taking any medications at all and reported having no illnesses, i.e., our control group. Future research should investigate this issue further.

The results of this study, if duplicated in future research on larger samples, could play a positive role in the care and effective management of diabetes in older women. According to the Health Belief Model described earlier [10], individuals tend to take action in order to take care of health problems once they believe that the problem is serious, based on their beliefs and attitudes related to health and treatment. However, as previously mentioned, with advanced age, attitudes and beliefs about health can become negative, while the ways in which individuals conceptualize the consequences of a serious health event or outcome, such as a diagnosis of diabetes, decrease in severity, with older adults attributing their health problems mainly to older age. The findings of the present study seem to somewhat contradict the Health Belief Model; this could be a by-product of the above-mentioned issues, but more research including ethnically diverse older women is certainly needed on this topic. Our findings support our hypothesis stating that older diabetic women have as stronger desire to improve their health. It is likely that these women perceived their diagnosis to be severe in nature, which was reflected in their strong desire to improve their health (regardless of their age). Again, more studies are needed in order to clarify the reasons for our research findings.

This investigation adds to the understanding of the cognitive function and health behaviors of diabetic older women; however, several limitations to the study exist. For instance, our sample was comprised exclusively of older women from Los Angeles County, which limits the generalizability of our findings. The use of self-report data also limited our study, as our data was subjective/not independently verified; however, most of the studies in this area were conducted using self-report measures as well. Also, the data in our study did not permit us to separately analyze type 1 versus type 2 diabetes, which may be differently associated with worse cognitive health outcomes. In addition, duration of diabetes could influence receipt of health screenings among older diabetic women, which, as already mentioned, was not evaluated in the present study. Finally, we did not examine the role of patient characteristics that may have affected receipt of health screenings such as lifestyle, obesity, and cultural factors.

## 5. CONCLUSIONS

As suggested by the findings of this preliminary investigation, when it comes to receiving health screenings and, at a marginally significant level, in regard to cognitive functioning, older diabetic women are faring better than older women who do not take any medications a nd report having no chronic health conditions. Furthermore, diabetic women intended to improve their health significantly more than their healthy counterpart. More research is certainly needed on diabetic older women from diverse ethnic backgrounds to corroborate the present pilot findings, which contribute to filling a gap in the existing medical literature. Our results contradict those of prior studies in which it was reported that routine preventive care such as regular health screenings and cancer screening are typically neglected by older adults living with diabetes. Given the modest sample size of our sample and the other limitations of this investigation, more research is needed to understand whether and how the treatment of older women with diabetes influences cognitive function.

## Figures and Tables

**Table 1:** Description of participants’ characteristics

Variables	Frequency	%	Range	M	SD
Age			50–90	69.29	8.175
**Married status**					
Yes	38	52.8			
No	34	47.2			
**Race/Ethnicity**					
Black/African American	5	6.9			
Asian American	11	15.3			
Mexican American	7	9.7			
Other Hispanic/Latino	7	9.7			
European American	34	47.2			
Other/Mixed	8	11.1			
**Education**					
Less than high school	13	18.1			
Graduated high school	18	25			
Completed trade school	8	11.1			
Some college	16	22.2			
Bachelor's degree	11	15.3			
Some graduate school	2	2.8			
Master's degree	3	4.2			
Ph.D., M.D., and/or J.D.	1	1.4			

**Table 2:** ANOVA results

	*F*	*η^2^*	*d*
*Wanting to Improve Health*			
Diabetes vs. no diabetes	11.87*	.15	.82

*Cognitive Functioning Scores*			
Diabetes vs. no diabetes	3.30	.05	.43

Note: Significance indicated by * p<.05

**Table 3:** Chi-square results

	*X^2^*	*p*	*d*
*Receiving Mammograms*			
Diabetes vs. no diabetes	5.87*	.015	.60

*Receiving Health Screenings*			
Diabetes vs. no diabetes	4.51*	.034	.52

Note: Significance indicated by * p<.05
